# Supplementary material for: A Critical View of the Application of the APEX Software (Aqueous Photochemistry of Environmentally-Occurring Xenobiotics) to Predict Photoreaction Kinetics in Surface Freshwaters
Source: Molecules. 2019 Dec 18;25(1):9. doi: 10.3390/molecules25010009 (PMC7017383; doi:10.3390/molecules25010009)
Supplement: Supplementary file 1 [file molecules-25-00009-s001.zip › 3.2.4_gcc-4.4.0.zip!/3.2.4_gcc-4.4.0/3.2.4_gcc-4.4.0/doc/octave/html/interpreter/Creating-a-Class.html]

Creating a Class - Untitled


Next: Manipulating Classes,
Up: Object Oriented Programming


---

### 33.1 Creating a Class

We use in the following text a polynomial class to demonstrate the use
of object oriented programming within Octave. This class was chosen as
it is simple, and so doesn't distract unnecessarily from the
discussion of the programming features of Octave. However, even still
a small understand of the polynomial class itself is necessary to
fully grasp the techniques described.

The polynomial class is used to represent polynomials of the form

```
     a0 + a1 * x + a2 * x^2 + ... + an * x^n
```

where
a0, a1, etc. are real scalars.
Thus the polynomial can be represented by a vector

```
     a = [a0, a1, a2, ..., an];
```

We therefore now have sufficient information about the requirements of
the class constructor for our polynomial class to write it. All object
oriented classes in Octave, must be contained with a directory taking
the name of the class, prepended with the @ symbol. For example, with
our polynomial class, we would place the methods defining the class in
the @polynomial directory.

The constructor of the class, must have the name of the class itself
and so in our example the constructor with have the name
@polynomial/polynomial.m. Also ideally when the constructor is
called with no arguments to should return a value object. So for example
our polynomial might look like

```
```
     ## -*- texinfo -*-
     ## @deftypefn {Function File} {} polynomial ()
     ## @deftypefnx {Function File} {} polynomial (@var{a})
     ## Creates a polynomial object representing the polynomial
     ##
     ## @example
     ## a0 + a1 * x + a2 * x^2 + @dots{} + an * x^n
     ## @end example
     ##
     ## from a vector of coefficients [a0 a1 a2 ... an].
     ## @end deftypefn
     
     function p = polynomial (a)
       if (nargin == 0)
         p.poly = [0];
         p = class (p, "polynomial");
       elseif (nargin == 1)
         if (strcmp (class (a), "polynomial"))
           p = a;
         elseif (isvector (a) && isreal (a))
           p.poly = a(:).';
           p = class (p, "polynomial");
         else
           error ("polynomial: expecting real vector");
         endif
       else
         print_usage ();
       endif
     endfunction
```
```

Note that the return value of the constructor must be the output of
the `class` function called with the first argument being a
structure and the second argument being the class name. An example of
the call to this constructor function is then

```
     p = polynomial ([1, 0, 1]);
```

Note that methods of a class can be documented. The help for the
constructor itself can be obtained with the constructor name, that is
for the polynomial constructor `help polynomial` will return the
help string. Also the help can be obtained by restricting the search
for the help to a particular class, for example `help
@polynomial/polynomial`. This second method is the only means of
getting help for the overloaded methods and functions of the class.

The same is true for other Octave functions that take a function name
as an argument. For example `type @polynomial/display` will
print the code of the display method of the polynomial class to the
screen, and `dbstop @polynomial/display` will set a breakpoint
at the first executable line of the display method of the polynomial
class.

To check where a variable is a user class, the `isobject` and
`isa` functions can be used. for example

```
     p = polynomial ([1, 0, 1]);
     isobject (p)
     => 1
     isa (p, "polynomial")
     => 1
```

— Built-in Function: **isobject** (x)  
> Return true if x is a class object.

The available methods of a class can be displayed with the
`methods` function.

— Built-in Function: **methods** (x)  
— Built-in Function: **methods** ("classname")  
> Return a cell array containing the names of the methods for the
> object x or the named class.

To inquire whether a particular method is available to a user class, the
`ismethod` function can be used.

— Built-in Function: **ismethod** (x, method)  
> Return true if x is a class object and the string method
> is a method of this class.

For example

```
     p = polynomial ([1, 0, 1]);
     ismethod (p, "roots")
     => 1
```
